# Comorbidity-Guided Text Mining and Omics Pipeline to Identify Candidate Genes and Drugs for Alzheimer’s Disease

**DOI:** 10.3390/genes15050614

**Published:** 2024-05-11

**Authors:** Iyappan Ramalakshmi Oviya, Divya Sankar, Sharanya Manoharan, Archana Prabahar, Kalpana Raja

**Affiliations:** 1 Department of Computer Science and Engineering, Amrita School of Computing, Amrita Vishwa Vidyapeetham, Chennai 641112, India; ir_oviya@ch.amrita.edu; 2 Department of Sciences, Amrita School of Engineering, Amrita Vishwa Vidyapeetham, Chennai 601103, India; s_divya@ch.students.amrita.edu; 3 Department of Bioinformatics, Stella Maris College, Chennai 600086, India; sharanyas.m@stellamariscollege.edu.in; 4 Center for Gene Regulation in Health and Disease, Department of Biological, Geological, and Environmental Sciences (BGES), Cleveland State University, Cleveland, OH 44115, USA; a.prabahar@csuohio.edu; 5 School of Biomedical Informatics, University of Texas Health Science Center, Houston, TX 77030, USA; 6 Section for Biomedical Informatics and Data Science, School of Medicine, Yale University, New Haven, CT 06510, USA

**Keywords:** Alzheimer’s disease, comorbid diseases, text mining, candidate gene identification, omics techniques, drug perturbation

## Abstract

Alzheimer’s disease (AD), a multifactorial neurodegenerative disorder, is prevalent among the elderly population. It is a complex trait with mutations in multiple genes. Although the US Food and Drug Administration (FDA) has approved a few drugs for AD treatment, a definitive cure remains elusive. Research efforts persist in seeking improved treatment options for AD. Here, a hybrid pipeline is proposed to apply text mining to identify comorbid diseases for AD and an omics approach to identify the common genes between AD and five comorbid diseases—dementia, type 2 diabetes, hypertension, Parkinson’s disease, and Down syndrome. We further identified the pathways and drugs for common genes. The rationale behind this approach is rooted in the fact that elderly individuals often receive multiple medications for various comorbid diseases, and an insight into the genes that are common to comorbid diseases may enhance treatment strategies. We identified seven common genes—*PSEN1*, *PSEN2*, *MAPT*, *APP*, *APOE*, *NOTCH*, and *HFE*—for AD and five comorbid diseases. We investigated the drugs interacting with these common genes using LINCS gene–drug perturbation. Our analysis unveiled several promising candidates, including MG-132 and Masitinib, which exhibit potential efficacy for both AD and its comorbid diseases. The pipeline can be extended to other diseases.

## 1. Introduction

Alzheimer’s disease (AD), a neurological disease that commonly affects elderly individuals, is characterized by a progressive decline in cognitive function and memory [1]. The National Institute on Aging estimates that more than six million people aged 65 or older are affected with AD in the United States (US). Current therapeutic interventions for AD strive to alleviate symptoms and potentially delay the progression of the condition. However, discovering a cure for the disease remains challenging due to its intricate nature, influenced by variations within multiple genes and affected by genetic and environmental factors [2]. Recently, the US Food and Drug Administration (FDA) has approved Aducanumab, a human antibody for treating early-stage AD. The medication targets the β-amyloid protein to mitigate the amyloid plaques or brain lesions associated with AD. The FDA also approved donepezil, rivastigmine, and a combination of donepezil and memantine for treating moderate to severe AD. On 6 January 2023, the FDA approved Leqembi (lecanemab-irmb) for treating AD (https://www.fda.gov/, accessed on 6 May 2024). Despite the available treatments for AD, researchers continue to seek improved treatment options.

Elderly patients are generally diagnosed with multiple diseases and they receive simultaneous treatment for all. Knowledge on these comorbid diseases and related treatments is vital when exploring the treatment options for diseases such as AD [3]. The treatments administered for comorbid diseases may impact AD because the medications prescribed for AD and its comorbid diseases may interact with each other. Most of the existing works on identifying the comorbid diseases for a specific disease rely on the patients’ electronic health records (EHRs) [4,5]. However, access to EHR data is not readily available. Obtaining permission from an Institutional Review Board (IRB) is necessary [6]. Alternatively, PubMed contains numerous biomedical articles related to comorbidity. A basic query to search comorbidity in the Medical Subject Heading (MeSH) index, “Comorbidity” [MeSH], retrieved 129,341 PubMed articles (accessed on 6 March 2024). The same basic query to search comorbidity in all sections of PubMed (e.g., Title, Abstract, and MeSH) retrieved 193,969 PubMed articles (accessed on 6 March 2024). Manual retrieval of comorbid diseases from PubMed is impractical and time-consuming. Alternatively, automated approaches such as text mining can process the entirety of PubMed and retrieve the needed information within a few minutes to hours. Unlike EHRs, which have numerous limitations on access, PubMed is freely accessible without any restrictions.

Recent advancements in high-throughput genomics, transcriptomics, and proteomics have driven the discovery of novel genes serving as biomarkers in diseases and targets of drug therapy [7]. With the advent of drug databases, computational screening of drugs is conducted using connectivity mapping methods, specifically CMap [8,9] and the Library of Integrated Network-based Cellular Signatures (LINCS) [10]. Both CMap and LINCS are comprehensive, large-scale drug perturbation databases with transcriptomic profiles at an unprecedented scale. CMap and LINCS have revolutionized drug discovery and systems biology by providing unprecedented access to large-scale, integrative datasets. These initiatives accelerate the translation of basic research findings into clinically relevant applications and advancing precision medicine. They explore the connections between drugs, diseases, and biological processes by identifying similarities in gene expression patterns induced by different perturbagens. The library of gene and drug profiles aids in understanding disease mechanisms and identifying new drugs for each disease. This is achieved by verifying whether the genes up-regulated in a disease are down-regulated after drug perturbation, and vice versa, based on the enrichment score [8,10,11]. In summary, these resources characterize the ‘signatures’ of gene expression changes induced by small molecules [12]. These drug perturbation signatures are utilized to determine the drugs associated with various diseases. The computational omics research and biomedical text mining approaches may help in elucidating the association between genes, drugs, and pathways and facilitating the identification of potential new drugs for AD and its comorbid diseases [3].

In the current study, we developed a hybrid pipeline to extract the comorbid diseases for AD from PubMed using a simple text mining approach and to find the common genes for AD and five comorbid diseases for AD—dementia, type 2 diabetes, hypertension, Parkinson’s disease, and Down syndrome—using omics approaches. We further retrieved the drugs interacting with these common genes using omics approaches. The main objective is to find new treatment options (i.e., common genes and drugs) for AD and its comorbid diseases. The comorbid diseases associated with AD were extracted from the MeSH index of PubMed. Our omics pipeline uses GeneWeaver [13] to identify the common genes for AD and its comorbid diseases; STRING [14] and the Reactome database [15] to understand the functional association and pathway enrichment analysis; and Sigcom LINCS [16] for drug perturbation analysis to examine the impact of drugs on the up-regulation and down-regulation of the identified common genes. The major contributions of this work are as follows:

(i) The proposed approach integrates text mining and genomics together to find common genes and drugs for AD and its comorbid diseases. The existing works either use text mining or genomics to find new treatment options for a disease. The existing works do not consider the comorbid diseases too. Thus, we advance the existing research methods for finding new treatment options.
(ii) The proposed approach queries PubMed abstracts to extract comorbid diseases for AD. The existing approaches extract the comorbid diseases for a disease of interest from patients’ electronic health records (EHRs). However, accessing EHRs requires institutional revenue board (IRB) approval. There are other challenges too. Unlike patients’ EHRs, PubMed is an open source and it is free to use.
(iii) A comorbidity-guided approach is proposed to identify new candidate genes and drugs for AD and its comorbid diseases. To our knowledge, the existing works on identifying new candidate genes and drugs for a disease, especially AD, do not consider its comorbid diseases. However, the knowledge on comorbid diseases, their candidate genes, and drugs prescribed for treating these comorbid diseases is important to avoid drug–drug interaction.

We believe that our approach may reduce the number of drugs being prescribed for AD and its comorbid diseases, and avoid possible drug–drug interactions between the drugs prescribed to each disease (AD and its comorbid diseases).

## 2. Materials and Methods

### 2.1. Text Mining Approach

While many existing studies on identifying comorbid diseases utilize patients’ electronic health records (EHRs), the current study proposed a simple text mining approach to extract the comorbid diseases associated with AD from PubMed. The text mining pipeline includes two steps: (i) creating a lexicon of MeSH diseases and (ii) applying the created lexicon to the MeSH index in PubMed to retrieve the comorbid diseases for AD. 

#### 2.1.1. Lexicon of MeSH Diseases 

A total of 10,043 MeSH diseases belonging to 22 disease categories were collected from https://www.ncbi.nlm.nih.gov/mesh/1000067 (accessed on 6 May 2024). After removing the duplicates, the collection included 4871 unique MeSH diseases. The diseases categorized under “Animal Diseases” were excluded from the lexicon.

#### 2.1.2. Retrieving Comorbid Diseases from PubMed

A total of 1208 PubMed articles (accessed on 22 November 2022) were retrieved from PubMed in PubMed format using the Boolean query “Alzheimer disease” [MeSH] AND “Comorbidity” [MeSH]. A Python script was developed to retrieve the PubMed ID (PMID) and MeSH index. The disease mentions in the MeSH index were identified using the lexicon of MeSH diseases. Certain PubMed articles include two or more disease mentions other than AD in the MeSH index. We retrieved all possible pairs of AD and co-occurring diseases from the MeSH index. The co-occurring diseases were ranked based on sort ratio (Equation (1)). We selected the top five diseases for subsequent omics analysis.

(1) $$\mathrm{Sort}\;\mathrm{ratio}=\frac{P_{eachPair}}{P_{all}}$$

Here, *P_eachPair_* corresponds to the number of PubMed articles containing both AD and a co-occurring disease, while *P_all_* corresponds to the number of PubMed articles containing AD and at least one co-occurring disease (i.e., 843 PubMed articles). All 843 PubMed articles include the MeSH term “Comorbidity”.

The co-occurrence of AD and a disease in the MeSH index alone cannot validate their comorbidity. The significant association between AD and a co-occurring disease, along with the MeSH term “Comorbidity”, can validate comorbidity. We performed the Fisher Exact Test (FET) to identify the significantly associated co-occurring diseases with AD. We obtained four counts to estimate FET *p*-values:

(i) Number of PubMed articles with AD and a co-occurring disease in the MeSH index;
(ii) Number of PubMed articles with the co-occurring disease in the MeSH index;
(iii) Number of PubMed articles with AD in the MeSH index;
(iv) Total number of PubMed articles (125,924, access date 22 November 2022) with the MeSH term “Comorbidity” in the MeSH index.

The following Boolean queries were used to retrieve the counts, (i), (ii), (iii), and (iv):

- Boolean query (a) for count (i): “Alzheimer disease” [MeSH] AND “Comorbidity” [MeSH] AND “CO-OCCURRING DISEASE” [MeSH];
- Boolean query (b) for count (ii): “CO-OCCURRING DISEASE” [MeSH] AND “Comorbidity” [MeSH] NOT “Alzheimer disease” [MeSH];
- Boolean query (c) for count (iii): “Alzheimer disease” [MeSH] AND “Comorbidity” [MeSH] NOT “CO-OCCURRING DISEASE” [MeSH];
- Boolean query (d) for count (iv): “Comorbidity” [MeSH].

To avoid the double counting of PubMed articles between counts (i) and (ii), the articles with AD were excluded (see Boolean query (b)). Similarly, to avoid the double counting of PubMed articles between counts (i) and (iii), the articles with co-occurring disease were excluded (see Boolean query (c)). The FET *p*-value for AD and a co-occurring disease summarizes the significance of comorbidity via the association reported in PubMed. Our approach identified 321 significant comorbid diseases for AD. Among these, the top five significant comorbid diseases were selected for further analysis using omics approaches: dementia, type 2 diabetes, hypertension, Parkinson’s disease, and Down syndrome.

### 2.2. Genomics Approach

#### 2.2.1. Identification of Common Genes for AD and Five Comorbid Diseases 

The genes common to AD and five comorbid diseases under study were investigated using Geneweaver, a web server for the integration and analysis of heterogeneous functional genomic data (https://geneweaver.org/, accessed on 6 May 2024). Geneweaver aggregates data from several integrated databases including Entrez, Unigene, and HUGO Gene Nomenclature Committee (HGNC). The genes associated with AD, dementia, type 2 diabetes, hypertension, Parkinson’s disease, and Down syndrome were considered to elucidate their involvement in shared pathways. The genes chosen for each disease were sourced from Tier I (Public Resource Grade) in Geneweaver. The attributes such as MeSH (Medical Subject Headings), Gene Ontology, Human Phenotype (HP) and genome-wide association studies (GWASs) were linked to the gene sets using the search tool provided. The intersection of genes common to AD and comorbid diseases were analyzed using GeneWeaver’s ‘HiSim graph’ tool, with homology excluded. 

#### 2.2.2. Protein–Protein Interaction from STRING Database 

A basic understanding of the interactions among the common genes for AD and five comorbid diseases as well as common genes with other genes is necessary to explore their roles in different diseases. We utilized the protein–protein interactions (PPIs) from the STRING database (https://string-db.org/, accessed on 6 May 2024) to interpret the interactions among the common genes for AD and five comorbid diseases, as well as other genes. In the STRING database, each PPI is assigned with a score, ranging from 0 to 1, to indicate the confidence level of interaction. These scores do not reflect the strength or specificity of the interaction. The STRING database utilizes k-means clustering to construct PPI networks, with the maximum number of interactions set to <10, and a cutoff for the combined score of interactions fixed at >0.4. The combined score is derived from two independent scores, namely the normal score and the transferred score. Here, the normal score is calculated from the data of the organism of interest and the transferred score is computed from data that are not originally observed in the organism of interest but are transferred by homology in some other organism [15].

#### 2.2.3. Enrichment Analysis for Pathways 

The Reactome database (https://reactome.org/, accessed on 6 May 2024) was utilized to explore the participation of genes in diverse biological pathways through gene-set analysis or quantitative pathway analysis. The gene list generated after Geneweaver analysis served as the input for pathway analysis. The tool integrates pathway identifier mapping, over-representation, and expression analysis. A threshold criterion of *p* ≤ 0.05 was applied to identify the most relevant genes associated with the comorbid diseases under study.

#### 2.2.4. Drugs from Library of Integrated Network-Based Cellular Signatures (Sigcom LINCS) 

**Figure 1:**
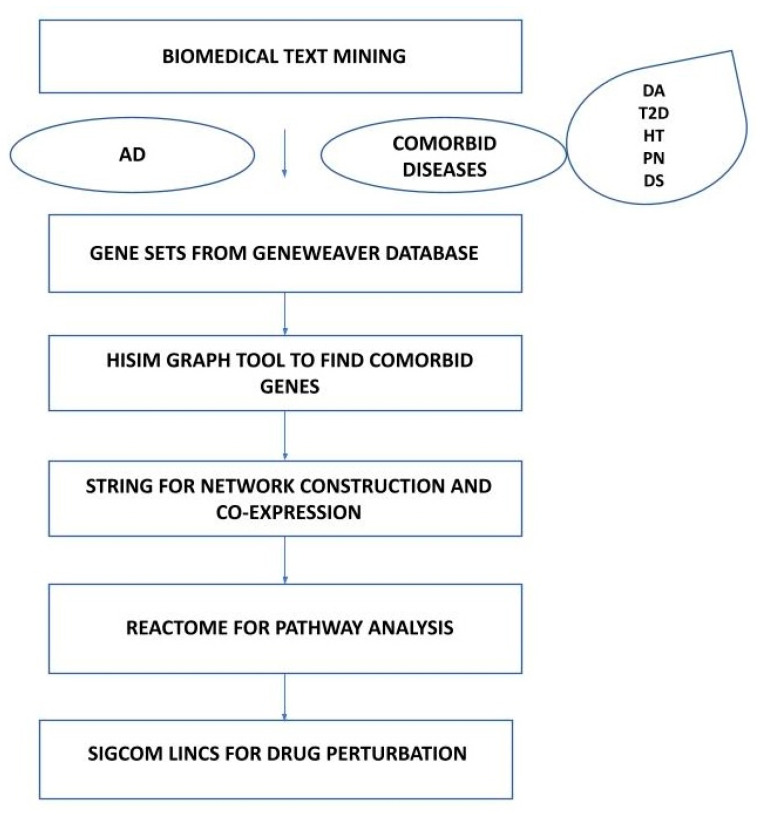
Workflow of genomics analysis for AD and five comorbid diseases. DA: dementia, T2D: type 2 diabetes, HT: hypertension, PN: Parkinson’s disease, DS: Down syndrome.

The genes shared between AD and each comorbid disease under study were the input to retrieve the interacting drugs from Sigcom LINCS, an online resource for accelerating drug and target discovery in systems pharmacology (https://maayanlab.cloud/sigcom-lincs/#/SignatureSearch/Set, accessed on 6 May 2024). Signature similarity searches were conducted and Sigcom LINCS chemical perturbations were leveraged to identify the relevant potential drugs. The approach provided insights into the mode of action for drugs, especially whether a drug up-regulates or down-regulates a given input gene. Subsequently, a gene–drug matrix was generated as a clustergram to visualize the expression of up-regulated and down-regulated genes. The overall genomic pipeline is illustrated in Figure 1.

## 3. Results

### 3.1. Comorbid Diseases for AD

Our text mining approach retrieved 321 co-occurring diseases for AD from 843 PubMed articles. Table 1 lists the top five co-occurring diseases for AD.

Among the top five co-occurring diseases, dementia, Parkinson’s disease, and Down syndrome are significantly associated with AD in PubMed. FET *p*-values lower than the traditional significance threshold value (<5.0 × 10^−8^) confirm this significant association. A manual search for at least one PubMed article validating the comorbidity between AD and each co-occurring disease provided evidence from the literature for all five co-occurring diseases (Table 2).

### 3.2. Common Genes among AD and Comorbid Diseases

Geneweaver retrieved 42 common genes for AD and dementia, five for AD and type 2 diabetes, 26 common genes for AD and hypertension, 22 common genes for AD and Parkinson’s disease, and 33 common genes for AD and Down syndrome (Supplementary Data S1). Dementia, being the most common comorbid disease for AD, has the maximum number of common genes. Interestingly, our approach retrieved only five common genes for AD and type 2 diabetes.

Table 3 shows the common genes for AD and two or more comorbid diseases. The results validate the potential multimorbidity among the diseases under study. The network analysis using the STRING database shows that most of the genes listed in Table 3 interact at a moderate level of confidence. A total of seven genes, *PSEN1*, *PSEN2*, *MAPT*, *APP*, *APOE*, *NOTCH2*, and *HFE*, are the most promising ones, as they were found to be involved in multimorbidity, making them potential targets. Our study provides insight into the genetic interplay underlying the development of AD in the presence of other comorbid diseases and enhances the understanding of the molecular basis of the disease.

### 3.3. Network Analysis of Common Genes

The analysis of gene interactions using the STRING database reveals a significant level of interdependency among the common genes, emphasizing their pivotal role in the comorbid diseases under study. In the STRING database, each protein–protein interaction (PPI) is annotated with one or more ‘scores’, which range between 0 and 1, indicating the confidence level. For common genes, most of the PPIs were reported with high confidence (scores > 0.8). This validates the comorbidity between AD and the five diseases under study through gene interaction and co-expression (Supplementary Data S2). The depth of the color in Supplementary Data S2 highlights the intensity of the co-expression analysis. Table 4 summarizes the common genes, pathways, and PPI enrichment for AD and each comorbid disease under study. The nature of interactions among the genes involved in comorbid diseases is justified with the analysis using the STRING database (Supplementary Data S3).

**Figure 2:**
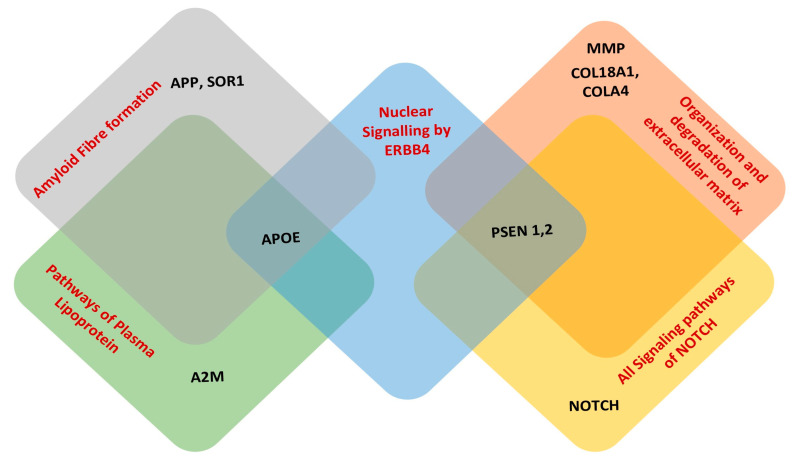
Interacting genes across different pathways.

The intersection of genes across diverse disease pathways underscores the gaps in the research focused on comprehending the triggering components in the onset of AD. Though there are multiple factors involved, the genetic mutations and variations in the genes are among the most significant factors. Understanding the underlying causes of these variations will pave the way for research avenues into the regulation of AD. Pathway analysis was conducted for 26 genes which were found to be common among the comorbid diseases, and functional profiling was carried out using g:Profiler, a web-based tool for further analysis. The analysis finds the ontologies from three perspectives, namely molecular function, cellular component, and biological processes. In a further analysis, the overlapping genes among the interpreted GO functions were established (Figure 2). This may shed light on novel target genes and pathways that can be explored to treat comorbidity. This approach can significantly reduce the prescription and usage of multiple drugs and their related side effects. 

### 3.4. Comorbid Diseases and Drug Perturbation

We further analyzed the common genes for drug perturbation using Sigcom LINCS. The drug perturbation signatures generated using Sigcom LINCS highlight the connections between the drugs, dosage, and their effect on gene expression. Table 5 shows the top 10 drugs, ranked based on *p*-value, predicted by Sigcom LINCS for AD and each of its comorbid diseases under study. The predicted *p*-value shows the significance of the drug for AD and its comorbid disease via the common genes. Supplementary Data S4 shows the interaction between common genes identified for AD and each comorbid disease under study and drugs and the role of the top 10 drugs in the up-regulation or down-regulation of genes for AD and its comorbid diseases under study. We also conducted the perturbation analysis of genes involved in multimorbidity among AD, type 2 diabetes, hypertension, Parkinson’s disease, and Down syndrome to find the common drugs that can target common pathways.

## 4. Discussion

AD is an age-related progressive disorder with a complex pathology. The disease is histopathologically characterized by the presence of extracellular amyloid β (Aβ) plaques and intracellular neurofibrillary tangles. Symptomatically, it manifests as gradual and progressive memory loss that impacts the individual’s personality. Over time, numerous research studies have contributed to the understanding of the genetic factors underlying the disease, which involve dysfunction in various genes such as *PSEN1*, *PSEN2*, *PRNP*, *APP*, *APOE*, *CASP7*, *MAPT*, *ABCA1*, *ABCA7*, *SORL1*, *RAB10*, *PICALM*, *TREML2*, and others [17]. These genes also serve as viable disease-modifying targets for AD, offering the opportunity for modulation during and/or after pathological onset, but before cognitive impairment. Mendelian mutations in genes like *APP*, *PSEN1*, and *PSEN2* are demonstrated to directly influence AD [18]. The current analysis corresponds to a similar examination of genes associated with AD and their variants, as summarized in earlier studies [19]. The present study also corroborated the association between AD and its comorbid diseases such as dementia, Parkinson’s disease, and type 2 diabetes, as documented earlier [20,21,22,23,24,25,26].

### 4.1. Comorbid Diseases and Genetic Analysis

*APP*, *APOC1*, *APOE*, *SORL1*, and *MAPT* are highly relevant common genes for AD and dementia. The combined role of *APP* and *APOE* in AD and dementia is highlighted in [27]. *APOE* (Apolipoprotein) is a major lipid transporter in the liver and brain, playing structural, regulatory, and functional roles in the repair and maintenance of the central nervous system (CNS). In humans, three allelic forms of the *APOE* gene are found, viz. ε2, ε3, and ε4 alleles. Variations in the ε4 allele are associated with an increased risk of AD. This allele is directly involved in cholesterol delivery and synthesis in the brain, regulated through an *APOE*-dependent mechanism, and plays a crucial role in regulating various signaling pathways [28]. The *Homeostatic Iron Regulator* (*HFE*) gene (*the hemochromatosis gene*) is responsible for transporting and regulating iron in the brain. While variations in HFE genes have been found to have a weak contribution to the genetic association with AD, their significance cannot be ignored. The present analysis reveals that *HFE* is implicated in multimorbidity with AD, dementia, hypertension, and Parkinson’s disease [29,30]. Many of the genes listed in Table 3 implicated comorbidity in the analysis, which aligns with previous research findings [19].

The gene encoding *Amyloid Precursor Protein* (*APP*) is located on Chromosome 21, and this protein plays a key role in the secretion of Aβ. This process is regulated by *PSEN1*. Elevated levels of Aβ are classic signs of AD, often resulting from mutations in the *APP* gene. This study observed an overlap of more than 30 genes between AD and dementia. An intriguing observation is that trisomy 21 also significantly contributes to an increased dosage effect of the *APP* gene, leading to AD and dementia [31,32].

The *APOE4* pathway influences *APP* signaling and recycling, leading to altered MAPK signaling and subsequent accumulation of Aβ. Additionally, *MAPT* plays a cascading role in disrupting intracellular calcium homeostasis and inhibiting *IST1* expression, contributing to cellular dysfunctions and neurodegenerative disorder [33]. Studies have demonstrated that *APOE4* can act as a signaling molecule, enhancing the activity of this cascade, and consequently increasing the levels of *APP*. *Presenilin-1* (*PSEN-1*) and *Presenilin-2* (*PSEN-2*) are homologous genes located on different chromosomes. Mutations in *PSEN-1* are high and have a direct effect on Aβ levels, whereas mutations in *PSEN-2*, although rare, still significantly disrupt γ-secretase activity, affecting the Aβ-42 and Aβ 42/40 ratio levels. Variants in *APP*, *PSEN1*, and *PSEN2* are involved in the production of amyloid β in rare autosomal dominant forms of early-onset AD [34]. Important sites of mutations in *APP* and associated defective functions related to AD pathogenicity are discussed [27]. 

Our analysis validates the interdependence of AD, dementia, and Parkinson’s disease through various common genes (i.e., *A2M*, *ABCA7*, *PLAU*, *PSEN2*, and *MPO*). This was also highlighted in an earlier study [23]. Both *PSEN-1* and *PSEN-2* are crucial in regulating NOTCH signaling, which itself is significant in the context of neurodegenerative diseases [35]. Notch proteins are vital for cell–cell signaling pathways, particularly during embryonic cell development and cell self-renewal systems. It has been established that dysfunctional Notch signaling pathways play a critical role in neurodegenerative diseases. The pathway is activated by five Notch ligands encoded by *JAG1*, *JAG2*, *DLL1*, *DLL3*, and *DLL4*, and four transmembrane receptors encoded by Notch genes (*NOTCH14*) [36]. *NOTCH2* is a common gene among AD, type 2 diabetes, and hypertension. Following the ligand–receptor interaction, the Notch molecules undergo proteolytic cleavage catalyzed by metalloprotease and γ-secretase. Subsequently, the Notch intracellular domain fragment is translocated to the nucleus, where it acts as a potent transcription activator, regulating processes like cell proliferation, differentiation, and apoptosis [37]. *PSEN1* variants have been reported to have a pleiotropic effect in Parkinson’s disease, another neurodegenerative disease that has also been demonstrated to be influenced by *PSEN1* variants [37].

In the current study, only five genes were found to be in common between AD and type 2 diabetes. However, genes such as *NOTCH2*, *LAMA1*, *TMEM94*, *TCF7L2*, and *CDKAL1* are found to be involved in AD and other diseases [20,38]. Members of the transmembrane (*TMEM*) protein family and their variants are also associated with Parkinson’s disease and other neurodegenerative diseases [38]. Similarly, *LAMA1* is implicated in dementia, providing a compelling rationale to interrogate its correlation with type 2 diabetes. An earlier study [20] reported that type 2 diabetes is correlated with AD and Parkinson’s disease. Type 2 diabetes, AD, and Parkinson’s disease are predicted to share similar dysregulated pathways involving *TCF7L2* and *CDKAL1* [20].

*Sortilin-related receptor1* (*SORL1*/*LR11*/*SORLA*) is a transmembrane protein involved in the intracellular sorting and trafficking of proteins into their respective subcellular compartments. The level of *SORL1* is reduced in AD patients with elevated Aβ due to several genetic alterations, which affects *APP*, *APOE*, and tau protein [39]. The analysis revealed that *SORL1* is associated with AD, Parkinson’s disease, and dementia. Mutations in *SORL1* have a functional role in both early-onset and late-onset AD [22]

The *NOS3* (*Nitric Oxide Synthase 3*, *Endothelial Cell*) gene was identified to be involved in AD, dementia, Parkinson’s disease, and hypertension. This highlights the role of *NOS3* in both comorbidity and multimorbidity. Variations in the *NOS3* gene, which regulates vascular tone, have been linked to susceptibility to coronary spasm, a condition associated with the onset of AD [40]. Polymorphisms in the *NOS3* and *nitric oxide synthase* (*NOS2A*) gene have been suggested to lead to an increased risk of AD. *NOS3* is identified as a comorbid gene in both AD and dementia. However, no substantial correlation was found between *APOE* genotypes affecting the *NOS3* genotype in AD and dementia [41].

With this analysis, it can be concluded that identifying early onset is crucial for disease management, which can be achieved through an understanding of genes involved in comorbidity and multimorbidity among AD, dementia, hypertension, type 2 diabetes, Parkinson’s disease, and Down syndrome. Additionally, bootstrapping in Geneweaver may further refine the search. By default, the condition was set as FALSE for analysis. However, when the bootstrapping condition was set to “TRUE”, the *APOE* gene was emphasized in comorbidity between AD and T2D. This finding aligns with other reports in the literature where *APOE* is indicated as a disease-causing gene [42,43]. 

### 4.2. Comorbid Diseases and Pathway Analysis

The STRING database illustrates the interplay of genes involved in the comorbidity with other genes through PPI network. The interaction and co-expression of genes are shown in Supplementary Data S2. The associated pathways were mapped to the genes using Reactome (https://reactome.org/, accessed on 6 May 2024). The inconsistent PPIs from the dataset were removed by defining the confidence interaction score threshold of ≥0.4. The gene enrichment was analyzed using the Bioconductor R package, ReactomePA, focusing on pathways with a significance threshold (*p*-value) of <0.05. The top scoring pathways were identified for further study. Specifically, the Reactome results highlighted the pathways pertaining to *NOTCH (1–4)* signaling, the organization and degradation of extracellular matrix signaling, nuclear signaling by *ERBB4*, amyloid fiber formation, and pathways of plasma lipoprotein. 

These pathways are interconnected, with few genes intersecting across them, as illustrated in Supplementary Data S2. *PSEN1* and *PSEN2* are the common genes involved in *NOTCH (1–4)* signaling, the organization and degradation of the extracellular matrix, and nuclear signaling by *ERBB4*. On the other hand, *APOE* is a common gene in nuclear signaling by *ERBB4*, amyloid fiber formation, and pathways of plasma lipoprotein. *PSEN1*, *PSEN2*, and *APOE* are linked via nuclear signaling pathways. The combined role of *PSEN* and *NOTCH* has been discussed earlier, and these genes are involved in signaling across all Notch pathways, including the activation and transmission of signals to the nucleus. The organization and degradation of the extracellular matrix pathway involves *PSEN*, which is influenced by the expression of *COL* and *MMP*. It is noteworthy that the shortlisted genes in the current analysis (Supplementary Data S1) are among the most extensively studied genes in the AD pathway and in individual pathways of other diseases [19]. The genes *APOE*, *APP*, and *SORL1* participate in amyloid fiber formation, while A2M and *APP* are associated with pathways of plasma lipoprotein. Reactome pathway analysis helps in predicting the missing gene links contributing to comorbidity. 

### 4.3. Comorbid Diseases and Drug Perturbation

The common genes among the pairs of comorbid diseases were identified, and their drug perturbations were analyzed using Sigcom LINCS. The gene–drug matrix table was then utilized for further analysis. In the current study, about 117 drugs were identified to be common among AD and the comorbid diseases. MG-132, a proteasome inhibitor known to reduce the degradation of ubiquitin-conjugated proteins in mammalian cells, is found to be a potential drug for AD and dementia, AD and type 2 diabetes, AD and Parkinson’s disease, and AD and Down syndrome. Interestingly, our approach did not find MG-132 as a potential drug for AD and hypertension. Masitinib, a tyrosine kinase inhibitor (TKI) that targets the c-Kit pathway, was found to be a common drug for AD, dementia, type 2 diabetes, and Parkinson’s disease. The drug targets derived from drug perturbations were visualized for potential exploitation, as they act on common pathways shared by two or more diseases, thereby potentially reducing drug intake and associated side effects. GSK-461364, Salmeterol, Vandetanib, TW-37, SJB-shh-31, NVP-BEZ235, and Velnacrine were recognized as the common drugs for AD, dementia, and Parkinson’s disease. Drugs such as Quizartinib, Gefitinnib, ARRY-334543, SB-216763, and Cabozantinib were identified to be common for AD, type 2 diabetes, and Parkinson’s disease. Estramustine and Simvastatin were found to be common for AD, hypertension, and Parkinson’s disease. Torin-1 was the only drug found to be common for AD, Parkinson’s disease, and Down syndrome. We also observed that most of the drugs retrieved by our approach act as inhibitors of kinases, vascular endothelial growth factor receptor, and epidermal growth factor receptor. 

**Future work:** Analyzing a larger set of differentially expressed genes (DEGs) from RNAseq data could uncover new genes for AD. This approach may pave the way for new research avenues, leveraging a hybrid model that integrates biomedical text mining and genomics to enhance the understanding of comorbidity. 

## 5. Conclusions

The current analysis underscores AD as a multifactorial neurodegenerative condition, demonstrating comorbidity and multimorbidity with other diseases. The involvement of numerous genes in various pathways, alongside their distinct expression patterns, constitutes several significant factors contributing to AD onset. This insight can aid researchers and clinicians in profiling patients via their electronic health records (EHRs), facilitating the detection of susceptibility to AD at an early stage. Early interventions targeted at delaying disease progression could prove beneficial in promoting the healthier living of patients.

## Figures and Tables

**Table 1:** Top five diseases that co-occur with AD.

Co-Occurring Disease	PubMed Articles with AD and Co-Occurring Disease (P)	PubMed Articles with AD and All Co-Occurring Diseases (T)	Sort Ratio (P/T)
Dementia	142	843	0.1684
Type 2 diabetes	95	843	0.1127
Hypertension	60	843	0.0712
Parkinson’s disease	51	843	0.0605
Down Syndrome	43	843	0.0510

**Table 2:** Comorbidity among top five co-occurring diseases for AD.

Comorbid Disease (CD)	PubMed Articles				FET *p*-Value	Literature Evidence (PMID)
	AD and CD	CD	AD	Comorbidity		
Dementia	142	220	1066	125,924	1.30 × 10^−238^	11406927
Type 2 diabetes	95	11,209	1113	125,924	0.68	28922161
Hypertension	60	6726	1148	125,924	0.59	33888050
Parkinson’s disease	51	683	1157	125,924	6.28 × 10^−30^	26820182
Down syndrome	43	346	1165	125,924	1.88 × 10^−34^	28009725

**Table 3:** Genes common to AD and comorbid conditions involved in multimorbidity.

Multimorbid Diseases	Number of Common Genes	Common Gene(s)
AD, dementia, Parkinson’s disease, Down syndrome	4	*MAPT*, *PSEN1*, *APP*, *APOE*
AD, dementia, Parkinson’s disease	5	*A2M*, *ABCA7*, *PLAU*, *PSEN2*, *MPO*
AD, dementia, Down syndrome	2	*COL18A1*, *SYNJ1*
AD, dementia, hypertension, Parkinson’s disease	2	*HFE*, *NOS3*
AD, Parkinson’s disease, Down syndrome	1	*SORL1*
AD, type 2 diabetes, hypertension	1	*NOTCH2*
AD, dementia, hypertension	1	*TGFB2*
AD, dementia, type 2 diabetes	1	*LAMA1*

**Table 4:** Common genes, pathways, and PPI enrichment for comorbid diseases.

Comorbid Diseases	Common Genes	Pathways for Common Genes (Reactome) (*p* < 0.05)	PPI Enrichment (STRING) (*p*-Value)
AD, dementia	41	28	<1.0 × 10^−16^
AD, type 2 diabetes	5	36	3.56 × 10^−5^
AD, hypertension	26	33	3.33 × 10^−16^
AD, Parkinson’s disease	22	27	<1.0 × 10^−16^
AD, Down syndrome	33	92	<1.0 × 10^−16^

**Table 5:** Top 10 drugs predicted by Sigcom LINCS for AD and comorbid diseases.

AD and Comorbid Disease	Drug (Top 10)	*p*-Value
AD and Dementia	axitinib	2.19 × 10^−7^
	lamotrigine	8.11 × 10^−7^
	ataluren	1.99 × 10^−6^
	vandetanib	2.21 × 10^−6^
	etofylline	2.49 × 10^−6^
	BRD-K18100239	3.92 × 10^−6^
	AS-605240	4.53 × 10^−6^
	velnacrine	5.96 × 10^−6^
	BRD-K30758067	6.15 × 10^−6^
	quiflapon	7.10 × 10^−6^
AD and Type 2 Diabetes	BMS-777607	0.0021
	trifluoperazine	0.0022
	BRD-K18972207	0.0025
	pirenperone	0.0025
	lonidamine	0.0026
	ARRY-334543	0.0026
	BRD-K84094241	0.0027
	BRD-K89952884	0.0028
	CYT-997	0.0029
	roflumilast	0.0030
AD and Hypertension	vernakalant	5.73 × 10^−7^
	BRD-K40853697	1.38 × 10^−6^
	marimastat	1.64 × 10^−6^
	LXR-623	2.09 × 10^−6^
	regorafenib	2.54 × 10^−6^
	aclidinium	6.77 × 10^−6^
	α-estradiol	7.31 × 10^−6^
	BRD-K38373457	7.37 × 10^−6^
	pidotimod	7.95 × 10^−6^
	ifenprodil	8.46 × 10^−6^
AD and Parkinson’s disease	bortezomib	1.30 × 10^−6^
	maprotiline	2.52 × 10^−5^
	flupirtine	8.11 × 10^−5^
	PD-0325901	8.70 × 10^−5^
	salmeterol	9.98 × 10^−5^
	quizartinib	0.0001
	dapagliflozin	0.0001
	gefitinib	0.0001
	lomitapide	0.0002
	SB-216763	0.0002
AD and Down syndrome	BRD-A01960364	3.77 × 10^−8^
	MG-132	3.20 × 10^−7^
	BRD-A06909528	6.13 × 10^−7^
	teniposide	1.24 × 10^−6^
	MD-049	1.64 × 10^−6^
	BRD-A95820578	1.89 × 10^−6^
	pravastatin	5.51 × 10^−6^
	gatifloxacin	5.74 × 10^−6^
	VU-0418942-1	6.26 × 10^−6^
	marimastat	6.74 × 10^−6^

## Data Availability

The original contributions presented in the study are included in the article and Supplementary Materials, further inquiries can be directed to the corresponding author.

## Supplementary Materials

The following supporting information can be downloaded at https://www.mdpi.com/article/10.3390/genes15050614/s1: Supplementary Data S1: List of common genes predicted by Geneweaver for AD and its comorbid diseases under study. Supplementary Data S2: Gene interaction network and co-expression profiling for AD and each comorbid disease under study. Supplementary Data S3: Interaction of genes related to AD and each comorbid disease under study using STRING database. Supplementary Data S4: (A) Interaction between common genes identified for AD and each comorbid disease under study and drugs. (B) Top 10 drugs down-regulating and up-regulating the common genes for AD and each comorbid disease under study.
