# Supplementary material for: Comorbidity-Guided Text Mining and Omics Pipeline to Identify Candidate Genes and Drugs for Alzheimer’s Disease
Source: Genes (Basel). 2024 May 11;15(5):614. doi: 10.3390/genes15050614 (PMC11121575; doi:10.3390/genes15050614)
Supplement: Supplementary file 1 [file genes-15-00614-s001.zip › genes-2965344-supplementary Data S2.pdf]

Comorbidity-Guided Text Mining and Omics Pipeline to Identify Candidate Genes and Drugs for Alzheimer's Disease

Iyappan Ramalakshmi Oviya [1,†], Divya Sankar [2,†], Sharanya Manoharan [3], Archana Prabahar [4] and Kalpana Raja [5,6,*]

1 Department of Computer Science and Engineering, Amrita School of Computing, Amrita Vishwa Vidyapeetham, Chennai 641112, India; ir_oviya@ch.amrita.edu
2 Department of Sciences, Amrita School of Engineering, Amrita Vishwa Vidyapeetham, Chennai 601103, India; s_divya@ch.students.amrita.edu
3 Department of Bioinformatics, Stella Maris College, Chennai 600086, India; sharanyas.m@stellamariscollege.edu.in
4 Center for Gene Regulation in Health and Disease, Department of Biological, Geological, and Environmental Sciences (BGES), Cleveland State University, Cleveland, OH 44115, USA; a.prabahar@csuohio.edu
5 School of Biomedical Informatics, University of Texas Health Science Center, Houston, TX 77030, USA
6 Section for Biomedical Informatics and Data Science, School of Medicine, Yale University, New Haven, CT 06510, USA
* Correspondence: kalpana.rajaa@gmail.com
† These authors contributed equally to this work.

Supplementary Data S2. Gene interaction network and co-expression profiling for AD and each comorbid disease under study

Supplementary Data S2a. Gene interaction network and co-expression profiling for AD and dementia
Supplementary Data S2b. Gene interaction network and co-expression profiling for AD and type 2 diabetes
Supplementary Data S2c. Gene interaction network and co-expression profiling for AD and hypertension
Supplementary Data S2d. Gene interaction network and co-expression profiling for AD and Parkinson's disease
Supplementary Data S2e. Gene interaction network and co-expression profiling for AD and down syndrome

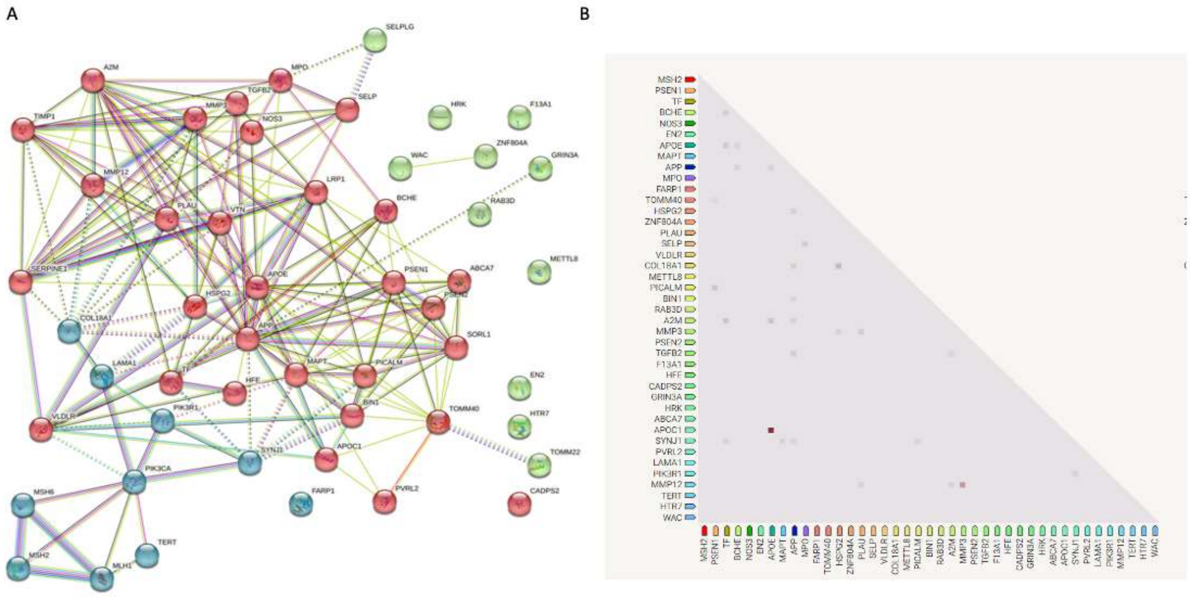

Supplementary Data S2a. Gene interaction network and co-expression profiling for AD and dementia

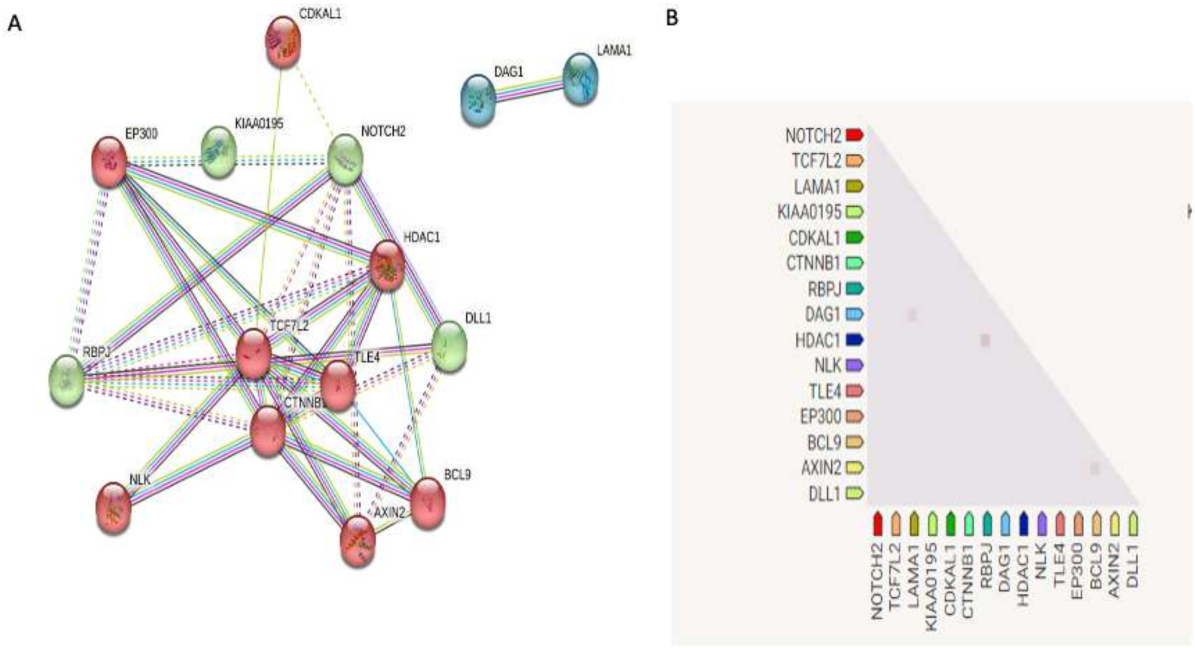

Supplementary Data S2b. Gene interaction network and co-expression profiling for AD and type 2 diabetes

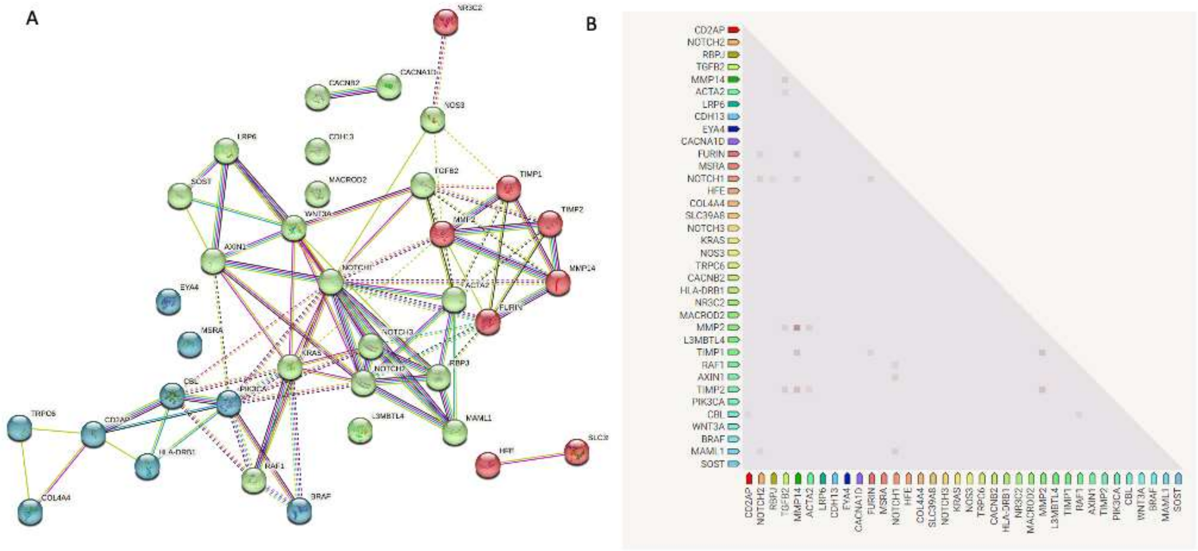

Supplementary Data S2c. Gene interaction network and co-expression profiling for AD and hypertension

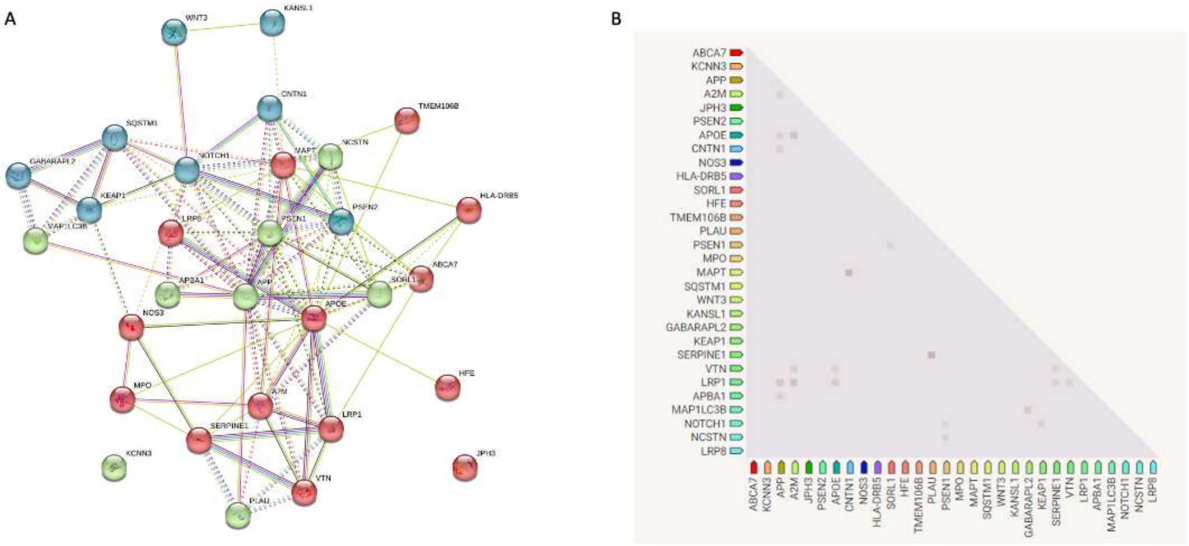

Supplementary Data S2d. Gene interaction network and co-expression profiling for AD and Parkinson's disease

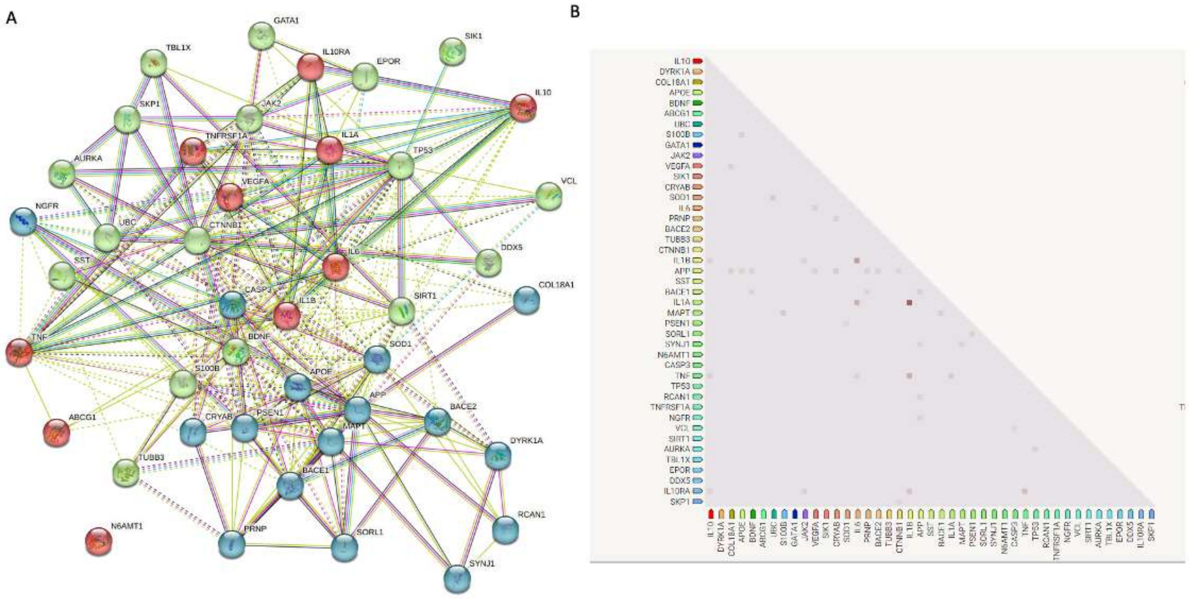

Supplementary Data S2e. Gene interaction network and co-expression profiling for AD and down syndrome
