# Supplementary material for: Comorbidity-Guided Text Mining and Omics Pipeline to Identify Candidate Genes and Drugs for Alzheimer’s Disease
Source: Genes (Basel). 2024 May 11;15(5):614. doi: 10.3390/genes15050614 (PMC11121575; doi:10.3390/genes15050614)
Supplement: Supplementary file 1 [file genes-15-00614-s001.zip › genes-2965344-supplementary Data S4.pdf]

Comorbidity-Guided Text Mining and Omics Pipeline to Identify Candidate Genes and Drugs for Alzheimer's Disease

Iyappan Ramalakshmi Oviya [1,†], Divya Sankar [2,†], Sharanya Manoharan [3], Archana Prabahar [4] and Kalpana Raja [5,6,*]

[1] Department of Computer Science and Engineering, Amrita School of Computing, Amrita Vishwa Vidyapeetham, Chennai 641112, India; ir_oviya@ch.amrita.edu
[2] Department of Sciences, Amrita School of Engineering, Amrita Vishwa Vidyapeetham, Chennai 601103, India; s_divya@ch.students.amrita.edu
[3] Department of Bioinformatics, Stella Maris College, Chennai 600086, India; sharanyas.m@stellamariscollege.edu.in
[4] Center for Gene Regulation in Health and Disease, Department of Biological, Geological, and Environmental Sciences (BGES), Cleveland State University, Cleveland, OH 44115, USA; a.prabahar@csuohio.edu
[5] School of Biomedical Informatics, University of Texas Health Science Center, Houston, TX 77030, USA
[6] Section for Biomedical Informatics and Data Science, School of Medicine, Yale University, New Haven, CT 06510, USA
* Correspondence: kalpana.rajaa@gmail.com
† These authors contributed equally to this work.

Supplementary Data S4. A. Interaction between common candidate genes identified for AD and each comorbid disease under study and drugs. B. Top 10 drugs downregulating and upregulating the common candidate genes for AD and each comorbid disease under study.

Supplementary Data S4a. A. Interaction between common candidate genes identified for AD and dementia and drugs. B. Top 10 drugs downregulating and upregulating the common candidate genes for AD and dementia.

Supplementary Data S4b. A. Interaction between common candidate genes identified for AD and type 2 diabetes and drugs. B. Top 10 drugs downregulating and upregulating the common candidate genes for AD and type 2 diabetes.

Supplementary Data S4c. A. Interaction between common candidate genes identified for AD and hypertension and drugs. B. Top 10 drugs downregulating and upregulating the common candidate genes for AD and hypertension.

Supplementary Data S4d. A. Interaction between common candidate genes identified for AD and Parkinson’s disease and drugs. B. Top 10 drugs downregulating and upregulating the common candidate genes for AD and Parkinson’s disease.

Supplementary Data S4e. A. Interaction between common candidate genes identified for AD and down syndrome and drugs. B. Top 10 drugs downregulating and upregulating the common candidate genes for AD and down syndrome.

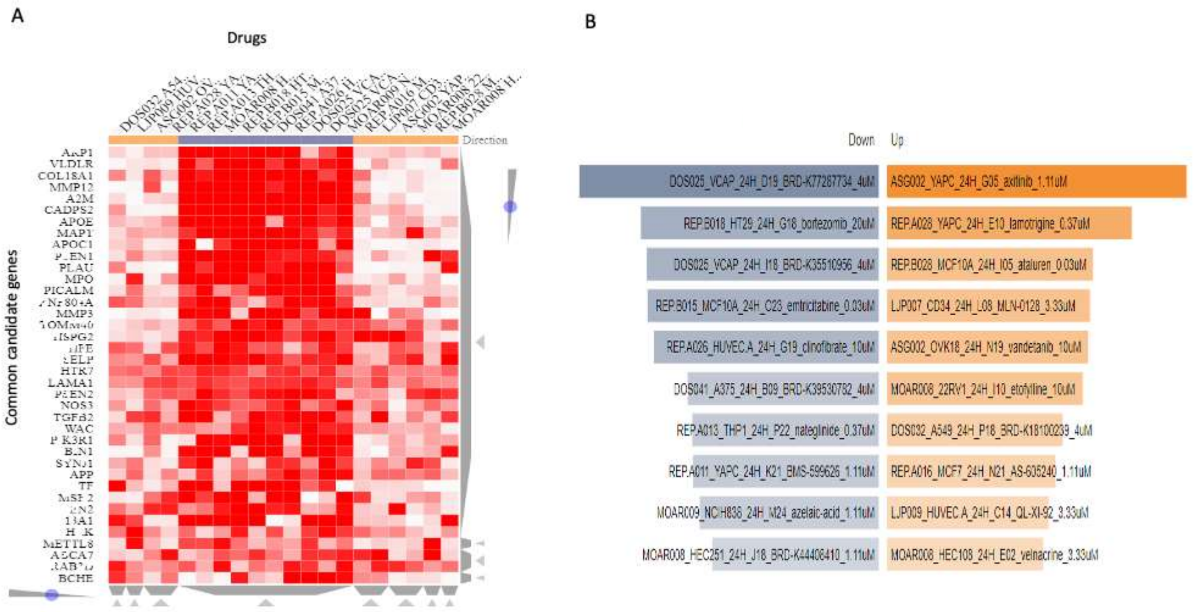

Supplementary Data S4a. A. Interaction between common candidate genes identified for AD and dementia and drugs. B. Top 10 drugs downregulating and upregulating the common candidate genes for AD and dementia.

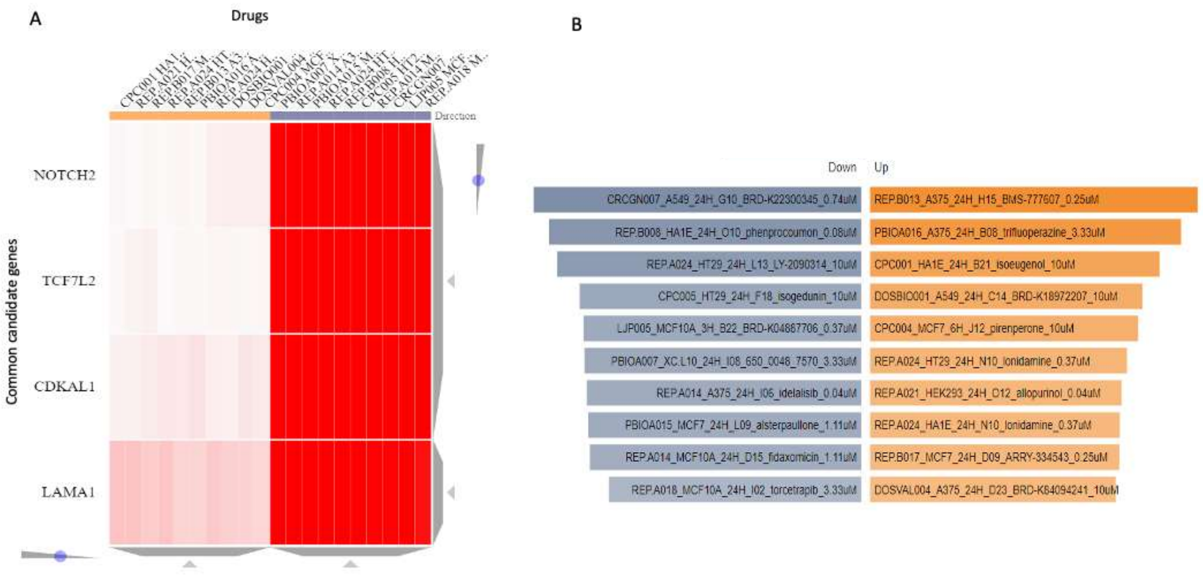

Supplementary Data S4b. A. Interaction between common candidate genes identified for AD and type 2 diabetes and drugs. B. Top 10 drugs downregulating and upregulating the common candidate genes for AD and type 2 diabetes.

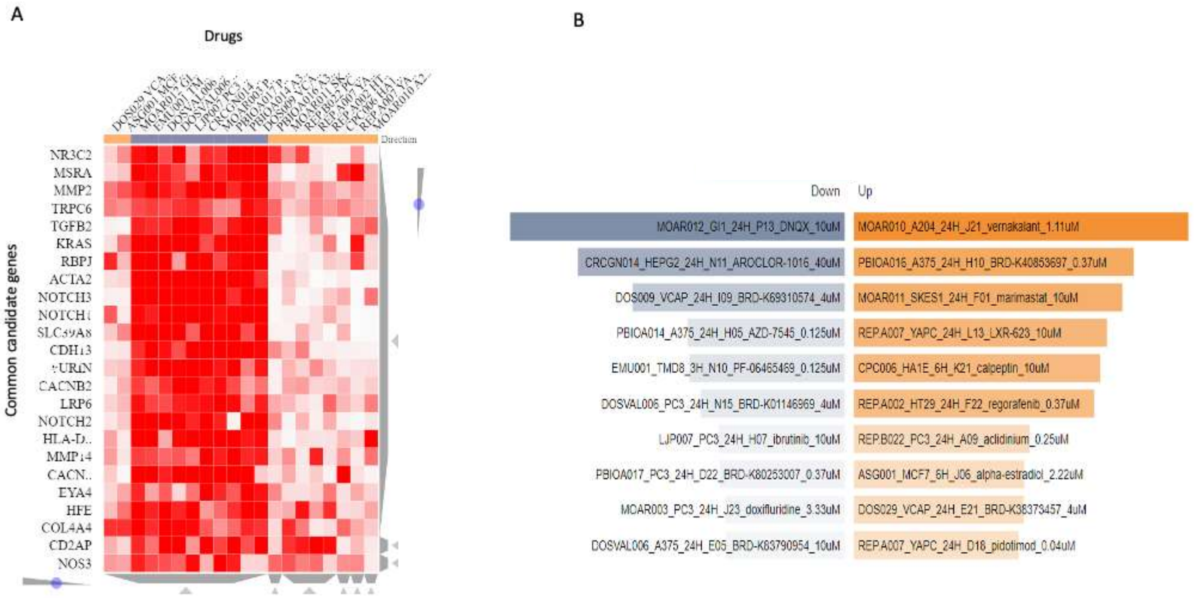

Supplementary Data S4c. A. Interaction between common candidate genes identified for AD and hypertension and drugs. B. Top 10 drugs downregulating and upregulating the common candidate genes for AD and hypertension.

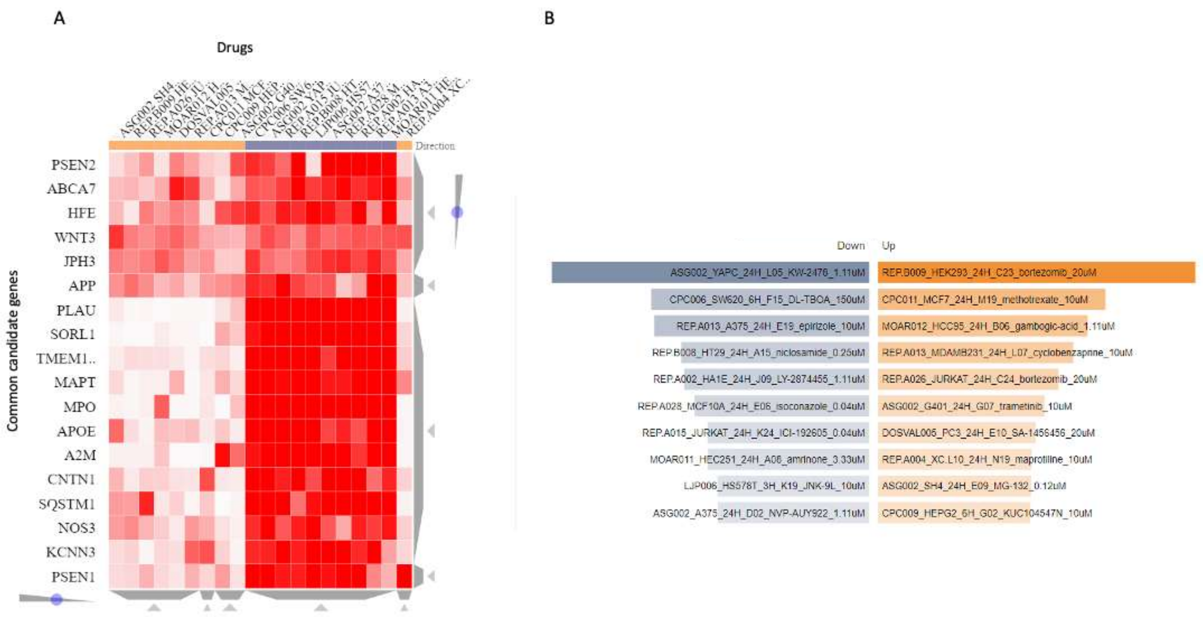

Supplementary Data S4d. A. Interaction between common candidate genes identified for AD and Parkinson's disease and drugs. B. Top 10 drugs downregulating and upregulating the common candidate genes for AD and Parkinson's disease.

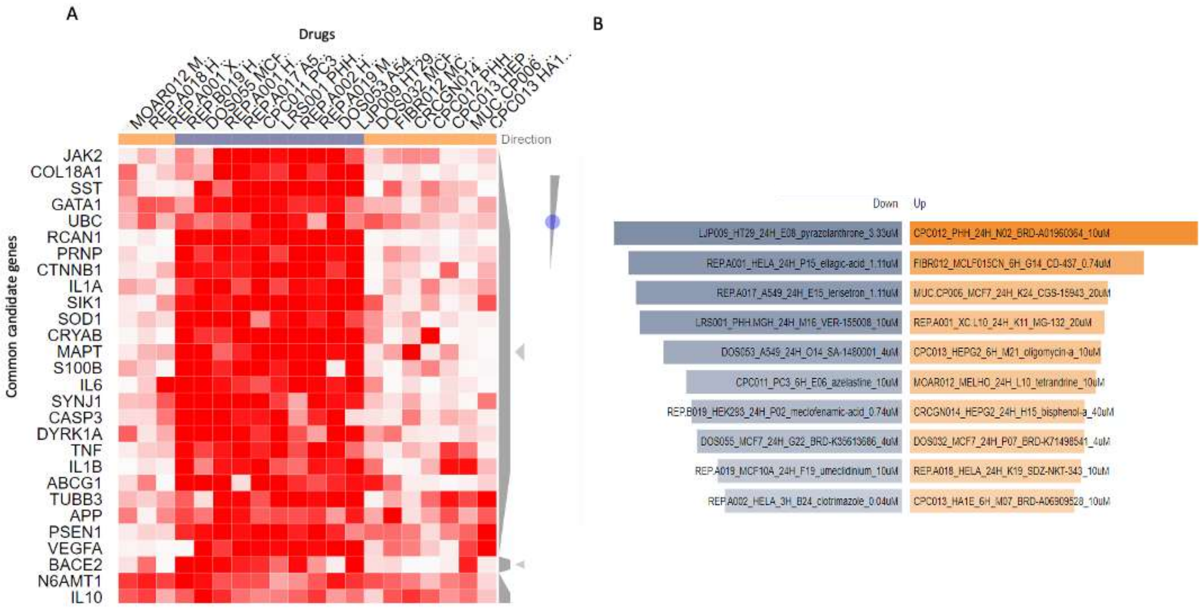

Supplementary Data S4e. A. Interaction between common candidate genes identified for AD and down syndrome and drugs. B. Top 10 drugs downregulating and upregulating the common candidate genes for AD and down syndrome.
